# Adult-Onset Gitelman Syndrome: Case Analysis and Literature Review

**DOI:** 10.1155/carm/2647228

**Published:** 2025-07-31

**Authors:** Intissar Haddiya, Sara Ramdani, Aymane Kadi, Imane Machmachi, Mohammed Benabdelhak, Yassamine Bentata

**Affiliations:** ^1^Department of Nephrology, Mohammed VI University Hospital, Faculty of Medicine and Pharmacy of Oujda, Mohammed First University, Oujda, Morocco; ^2^Laboratory of Epidemiology, Clinical Research and Public Health, Faculty of Medicine and Pharmacy of Oujda, Mohammed First University, Oujda, Morocco

**Keywords:** genetic disorder, Gitelman syndrome, hypokalemia, hypomagnesemia, kidney, metabolic alkalosis, *SLC12A3* gene, urine

## Abstract

**Background:** Gitelman syndrome is a rare autosomal recessive renal tubulopathy, characterized by hypomagnesemia, hypokalemia, hypochloremia, and metabolic alkalosis. The syndrome commonly presents with symptoms such as fatigue, muscle cramps, and tetany, impacting patients' quality of life. Although genetic confirmation via identification of mutations in the *SLC12A3* gene is ideal, resource constraints often limit access to these tests, especially in low-resource settings. This study aims to analyze the clinical, biochemical, and familial features of Gitelman syndrome in three patients, highlighting the syndrome's characteristic biochemical abnormalities and discussing the implications of limited genetic testing.

**Methods:** We conducted a comparative analysis of three diagnosed cases of Gitelman syndrome. Clinical presentations, biochemical data (with emphasis on magnesium and potassium levels), and family histories were systematically collected. Due to logistical limitations, genetic testing could not be performed. A comparative evaluation was then undertaken to assess commonalities and differences among the cases.

**Results:** All three patients presented with hallmark clinical features of Gitelman syndrome, including fatigue, muscle cramps, and intermittent tetany. Biochemical evaluation revealed persistent hypokalemia (serum potassium: 1.0–3.1 mmol/L), hypomagnesemia (0.53–0.60 mmol/L), and metabolic alkalosis (HCO_3_^−^: 28–31.5 mmol/L; pH: 7.40–7.45). Urinary electrolyte profiles demonstrated inappropriate renal losses of potassium (54 mmol/24 h), chloride (180–190 mmol/24 h), and sodium (70–120 mmol/24 h). Serum creatinine levels remained within normal limits (7–9.1 mg/L), and parathormone concentrations ranged from 30 to 32 pg/mL. No suggestive clinical signs of Bartter syndrome were observed, and secondary causes such as diuretic use, autoimmune nephropathies, and endocrinopathies were excluded. Family history was negative in two of the three cases, suggesting the potential for de novo mutations or undetected autosomal recessive inheritance. All patients were managed with oral potassium and magnesium supplementation, resulting in notable clinical and biochemical improvement, with follow-up serum potassium ranging from 3.5 to 3.9 mmol/L and magnesium from 0.74 to 1.3 mmol/L.

**Conclusion:** The clinical and biochemical findings in these patients are strongly indicative of Gitelman syndrome, even in the absence of genetic confirmation. This study emphasizes the necessity of a multidisciplinary approach in diagnosing and managing Gitelman syndrome, where biochemical assessments and clinical findings are instrumental. While genetic testing could provide conclusive evidence, effective management through electrolyte supplementation plays a crucial role in improving patients' quality of life.

## 1. Introduction

Gitelman syndrome is a rare genetic disorder characterized by hypokalemia, hypomagnesemia, and hypochloremic metabolic alkalosis. It is an inherited renal tubular disorder resulting from mutations in the *SLC12A3* gene, which encodes the thiazide-sensitive sodium-chloride cotransporter (NCCT) in the distal convoluted tubule (DCT) of the kidneys [1]. Gitelman syndrome is often diagnosed during adolescence or early adulthood, although earlier manifestations are possible [2].

In terms of epidemiology, the incidence of Gitelman syndrome is estimated to be approximately 1 in 40,000 individuals [3]. The disease appears to affect both sexes relatively equally, although some studies suggest a slight predominance in females [4].

The diagnosis of Gitelman syndrome is based on a combination of clinical, biochemical, and genetic criteria. Clinically, patients often present with nonspecific symptoms such as fatigue, muscle weakness, cramps, and sometimes episodes of periodic paralysis due to severe hypokalemia. Biochemical investigations typically reveal hypokalemia, hypomagnesemia, metabolic alkalosis, and hypochloremia. Low urinary calcium excretion is also a distinguishing feature compared to Bartter syndrome, another renal tubulopathy.

The diagnosis can be confirmed by genetic testing, identifying homozygous or compound heterozygous mutations in the *SLC12A3* gene. These tests are particularly useful in atypical cases or when symptoms are not sufficiently specific to differentiate Gitelman syndrome from other tubulopathies [5].

The treatment of Gitelman syndrome, in the absence of severe complications, primarily focuses on correcting electrolyte imbalances. This includes supplementation with potassium and magnesium to counter the deficiencies characteristic of the disease. Potassium-sparing diuretics, such as spironolactone or amiloride, can be used to reduce potassium loss.

In addition, a high-salt diet is often recommended to compensate for urinary sodium losses.

Early diagnosis and management of Gitelman syndrome are essential to prevent potential complications, improve patients' quality of life, and reduce recurrent hospitalizations due to severe electrolyte imbalances.

The objective of this paper was to report cases of Gitelman syndrome followed up in the nephrology department.

## 2. Patients and Methods

### 2.1. Study Type

Our study was conducted in the nephrology department of Mohammed VI University Hospital Center in Oujda, Morocco, over a period of 35 months between 2020 and 2023.

### 2.2. Inclusion Criteria

We included all patients aged 18 years and older, presenting with Gitelman syndrome, based on a combination of clinical and biochemical criteria.

The clinical criteria included typical symptoms such as chronic fatigue, muscle cramps, and episodes of tetany. Biochemically, the patients presented with hypokalemia, hypomagnesemia, and metabolic alkalosis, without known external etiologies requiring further investigations.

### 2.3. Exclusion Criteria

Patients who did not consent and those with incomplete or unusable medical records were excluded from the study.

### 2.4. Methodologies and Data Sources

Data collection was carried out from the patients' medical records using an exploitation sheet, analyzing the clinical, paraclinical, and therapeutic data of Gitelman syndrome. Articles and studies cited in the research were collected through searches on platforms such as Google Scholar, PubMed, Scopus, ResearchGate, Web of Science, Cochrane, and ScienceDirect, using keywords related to Gitelman syndrome.

Normal laboratory reference ranges used in this study were those provided by the central biochemistry laboratory of CHU Oujda. For adult patients, normal serum values were as follows: potassium 3.5–5.0 mmol/L, magnesium 0.70–1.05 mmol/L, chloride 98–107 mmol/L, calcium 2.15–2.55 mmol/L, phosphorus 0.81–1.45 mmol/L, sodium 135–145 mmol/L, bicarbonate 22–28 mmol/L, and arterial pH 7.35–7.45. Normal urinary excretion values over 24 h included potassium 25–125 mmol, sodium > 100 mmol, chloride 110–250 mmol, and calcium < 3.8 mmol.

### 2.5. Ethical Considerations

This study was conducted in accordance with the ethical principles outlined in the Declaration of Helsinki, ensuring the rights, safety, and well-being of the included patients, with respect for their anonymity and the confidentiality of their information. Moreover, informed written consent was obtained from each participant prior to their involvement.

## 3. Case Series

### 3.1. Case Study 1

A 25-year-old woman was admitted to the hospital in 2023 for investigation of hypokalemia.

Her history dates back to 2017, when she underwent a preoperative workup for cholecystectomy, where hypokalemia at 1 mmol/L with hypomagnesemia at 0.6 mmol/L and hypochloremia at 80 mmol/L were noted. The patient was hospitalized for potassium supplementation for 7 days, and then for oral potassium supplementation. However, hypokalemia persisted and she was referred to the nephrology department for investigation and management.

On clinical examination, the patient reported persistent vomiting, weight loss, and asthenia. She was hemodynamically stable with a blood pressure of 112/58 mmHg. She weighed 41 kg for a height of 156 cm, corresponding to a body mass index of 16.84 kg/m^2^, and no other significant features on physical examination were noted.

On laboratory evaluation, K^+^ = 3.1 mmol/L, Mg^2+^Na^+^ = 0.89 mmol/L, Ca^2+^ = 2.24 mmol/L, phosphorus = 1.13 mmol/L, Na = 140 mmol/L, and serum creatinine = 0.71 μmol/L were observed, as parathormone (PTH) = 30 pg/L, while pH = 7.40 and HCO_3_^−^ = 28 mmol/L. Autoimmune markers Anti-dsDNA and ANCA tests and hormone levels such as thyroglobulin, prolactin, cortisol, FSH, LH, anti-TPO, and anti-TG were reported within normal limits. 24 h urine Na = 115 mmol, Ca^2+^ = 5.0 mmol, and Cl^−^ = 180 mmol.

The diagnosis of Gitelman syndrome was established. Differential diagnoses were ruled out, especially Bartter syndrome Type 3, as the patient had no characteristic phenotype of Bartter syndrome (large eyes and triangular face), and use of drugs such as diuretics, laxatives, and cisplatin, as well as autoimmune diseases, was ruled out. Besides, other diagnoses were considered but not accepted, including primary hyperaldosteronism, pheochromocytoma, and hypercorticism, as no Cushing's syndrome, medication, or digestive disorders were noted. Conditions such as diabetes, polyuro–polydipsic syndrome, coarctation of the aorta, and obstructive sleep apnea syndrome were excluded.

Renal artery angioscan revealed normal-sized kidneys with patent renal arteries. Kidney ultrasound showed no structural abnormalities or nephrocalcinosis. Gitelman syndrome was suspected due to the combination of hypokalemia, hypomagnesemia, and hypochloremia. The patient was treated with oral potassium supplementation (10 tablets/day) and magnesium supplementation (1 ampoule/day). At the last follow-up, in May 2024, the results showed normal kalemia of 3.9 mmol/L and serum magnesium of 1.3 mmol/L.

### 3.2. Case Study 2

The patient was a 25-year-old woman admitted to the hospital in 2023 for the investigation and management of hypokalemia, which was initially identified during an episode of severe upper respiratory tract infection.

On clinical examination, she was alert, without respiratory distress, and showed hemodynamic stability, with a blood pressure of 120/60 mmHg and no signs of orthostatic hypotension. Her urine output was within normal limits at 1.6 L per 24 h, and there was no edema observed in the lower extremities. Urine dipstick tests were negative for hematuria, proteinuria, leukocytes, and nitrites, indicating no apparent urinary tract infection or renal disease markers.

The initial paraclinical evaluation upon admission revealed a blood creatinine level of 7 mg/L, severe hypokalemia of 2.1 mmol/L, hypomagnesemia of 0.53 mmol/L, phosphocalcic profile with a corrected serum calcium level of 95 mg/L and a phosphorus level of 34 mg/L, urinary 24 h potassium of 54 mmol and sodium of 70 mmol, and alkaline reserve of 31.5 mmol/L. PTH was at 30 pg/L and pH at 7.45.

The autoimmune markers (Anti-dsDNA and ANCA tests) and hormone levels (FSH, LH, cortisol, anti-TPO, anti-TG, thyroglobulin, and prolactin) were all reported within normal limits.

The diagnosis of Gitelman syndrome was established based on the presence of several hallmark clinical and biochemical findings, including hypokalemia, hypomagnesemia, elevated urinary potassium, and metabolic alkalosis, which are essential diagnostic indicators for this condition.

The patient's initial treatment involved oral potassium supplementation, administered as two tablets twice daily. She was discharged on this regimen and advised on dietary measures to support her treatment. Follow-up assessments revealed improved electrolyte levels, with a serum potassium of 3.5 mmol/L and a serum magnesium level of 0.74 mmol/L.

### 3.3. Case Study 3

The patient was a 30-year-old individual referred to our university hospital in 2020 for follow-up management of Gitelman syndrome. The initial diagnosis was made due to episodes of paralyzing hypokalemia, characterized by profound muscle weakness and paralysis, after excluding other potential causes of hypokalemia.

On admission, laboratory evaluations showed a serum potassium level of 4.2 mmol/L while on potassium supplementation, and serum creatinine was measured as 0.78 μmol/L. In addition, the evaluation revealed a serum magnesium level of 1.00 mmol/L, calcium of 2.37 mmol/L, phosphorus of 1.13 mmol/L, sodium of 140 mmol/L, and a PTH level of 32 pg/L. The patient's serum pH was 7.40, and HCO_3_^−^ was measured at 28 mmol/L. Anti-dsDNA, ANCA tests, and anti-TPO, anti-TG, cortisol, FSH, LH, thyroglobulin, and prolactin were all reported within normal limits.

Furthermore, the 24 h urine analysis revealed a sodium level of 120 mmol, calcium of 220 mg, and chloride of 190 mmol.

Five months later, the patient returned with symptoms of upper limb paresthesia. At this time, control laboratory results indicated a serum potassium level of 2.7 mmol/L. In response to the drop in potassium use and the appearance of paresthesia, the oral potassium supplementation dosage was increased.

Four months following this adjustment, the patient demonstrated notable clinical improvement, with resolution of upper limb paresthesia. Control laboratory values revealed a serum potassium level of 3.79 mmol/L and a serum creatinine level of 0.80 μmol/L. The treatment plan was to maintain oral potassium supplementation to stabilize the patient's condition.

## 4. Discussion

Patients with Gitelman syndrome presented with a variety of symptoms including persistent hypokalemia despite potassium supplementation. Treatment consisted mainly of potassium syrups and tablets, often adjusted according to serum levels and clinical symptoms. Patients also benefited from close monitoring of serum kalemia and magnesium, as well as other blood and urine tests. Interventions included intravenous rehydration and regular dosage adjustments to maintain adequate electrolyte levels. Long-term follow-up showed clinical and biochemical improvements, although ongoing treatment adjustments are often required to effectively manage the condition.

### 4.1. Diagnostic Approach to Gitelman Syndrome

Gitelman syndrome is a rare autosomal recessive tubulopathy caused by mutations in the SLC12A3 gene, affecting the NCCT in the DCT [6]. Also known as familial hypomagnesemia–hypokalemia with hypocalciuria [7], it impairs sodium reabsorption in the DCT, which normally accounts for 5%–10% of renal sodium handling [7, 8]. This defect leads to increased sodium delivery to the collecting duct, enhancing potassium and hydrogen loss, and causing hypokalemia and metabolic alkalosis. These imbalances are worsened by secondary hyperaldosteronism due to chronic salt loss.

Hypomagnesemia is primarily due to downregulation of the TRPM6 channel, expressed in the DCT [9], though some patients may have normomagnesemia, often associated with milder symptoms. The mechanism of hypocalciuria is unclear and likely multifactorial, with animal studies suggesting it is amplified under low-volume conditions during NCCT inhibition by thiazides [9, 10]. However, hypovolemia alone does not fully account for hypocalciuria, as volume expansion only partially reverses this effect. Studies in knock-in mouse models of Gitelman syndrome (with SLC12A3 mutations) suggest that increased expression of TRPV5 and TRPV6 channels in the DCT may also contribute [11]. Enhanced calcium reabsorption may explain the higher bone mineral density observed in these patients, similar to the effects of thiazide diuretics [12].

### 4.2. Clinical Presentation of Gitelman Syndrome

Gitelman syndrome is typically diagnosed in late childhood or early adulthood, though rare cases occur in the neonatal period or later in life. Symptoms are often nonspecific and may include muscle weakness, cramps, tetany, hypokalemic rhabdomyolysis, paralysis, hypotension, palpitations, and fatigue [13]. Children may show polyuria, polydipsia, muscle weakness, and growth retardation. Salt craving and a preference for salty foods are common clues, sometimes accompanied by a family history of similar traits.

Polyhydramnios is uncommon in Gitelman syndrome and more indicative of Bartter syndrome, which usually manifests earlier and presents with more severe salt wasting. Some patients with *CLCNKB* mutations exhibit overlapping features of both syndromes, as the encoded chloride channel is present in the thick ascending limb and the DCT [14]. In adults, Gitelman syndrome may present with complications such as chondrocalcinosis (linked to hypomagnesemia), glucose intolerance, or insulin resistance. Hypertension may develop later, potentially due to chronic hyperreninemia and juxtaglomerular hyperplasia from ongoing volume depletion [15].

The condition is not benign. Prolonged QTc intervals on ECG are found in about 50% of cases, and malignant arrhythmias have been reported. A baseline ECG is recommended for all patients, with further cardiologic evaluation if abnormalities persist despite electrolyte correction or if cardiac symptoms arise. In addition, many patients report a significant reduction in quality of life.

In our series, all three patients exhibited typical clinical manifestations of Gitelman syndrome, although variations in symptomatology were observed (Table 1).

• Case 1: The patient presented with persistent vomiting and a general deterioration in health, leading to thorough investigations to rule out a tumor. These symptoms, though nonspecific, were ultimately attributed to the typical electrolyte imbalances of Gitelman syndrome.
• Case 2: This patient initially exhibited more severe symptoms of paralytic hypokalemia, a clear indicator of Gitelman syndrome. Similar rare presentations have been reported in the literature, including a case of a 17-year-old male from Egypt who presented with recurrent lower limb paralysis due to severe hypokalemia, ultimately diagnosed as Gitelman syndrome [16].
• Case 3: Gitelman syndrome was already diagnosed in another facility, and potassium supplementation was prescribed. However, the drop in potassium use led to severe symptomatic hypokalemia. The clinical course showed a symptomatic response to treatment with an improvement in potassium levels and a reduction in symptoms.

### 4.3. Biochemical Presentation of Gitelman Syndrome

Gitelman syndrome is initially characterized by hypokalemia, hypomagnesemia, and metabolic alkalosis. However, serum magnesium levels can sometimes remain within normal ranges. Unlike Bartter syndrome, patients with Gitelman syndrome typically maintain a normal urine concentrating ability and show hypocalciuria. In contrast, Bartter syndrome often features hypercalciuria, nephrocalcinosis, and a reduced capacity for urine concentration due to impaired reabsorption in the loop of Henle [17].

To help distinguish between these two conditions, a urinary calcium/creatinine ratio is a useful marker, hypocalciuria (typically < 0.2, or < 0.6 mol/mol in broader definitions), which supports the diagnosis of Gitelman syndrome. Although a 24 h urine collection may be more precise, it is often limited by poor patient compliance and lacks strong evidence to justify routine use [18].

In Gitelman syndrome, urinary sodium and chloride excretion are typically elevated. A urinary sodium/chloride ratio close to 1 is indicative of a renal tubular origin. The fractional excretion of magnesium may also be increased, reflecting impaired fine-tuning by the DCT [1]. Chronic volume depletion often drives secondary hyperaldosteronism, which can occur with or without accompanying hypertension [19]. A clinical diagnosis of Gitelman syndrome requires the presence of persistent hypokalemia with inappropriately high renal potassium loss, typically alongside metabolic alkalosis. Due to the rarity and subtlety of symptoms, diagnosis is frequently delayed [20, 21].

In our series, the patients' biochemical data reflect the typical features of Gitelman syndrome, although variations were observed in potassium and magnesium levels (Table 1).

• Case 1: At presentation, the patient had a serum potassium level of 1 mmol/L, rising to 3.9 mmol/L after potassium supplementation, and then fluctuating. Serum magnesium was 0.6 mmol/L, indicating severe hypomagnesemia.
• Case 2: The patient initially had a serum potassium level of 2.1 mmol/L, necessitating potassium supplementation. Subsequent values showed improvement with a control potassium level of 3.5 mmol/L, in addition to metabolic alkalosis, with elevated alkaline reserves of 31.5 mmol/L.
• Case 3: The patient initially had a serum potassium level of 4.2 mmol/L after prior supplementation and a creatinine level of 88 μmol/L, with notable fluctuations during treatment. These variations in biochemical data necessitate constant adjustment of treatment to maintain electrolyte levels within acceptable ranges.

### 4.4. Differential Diagnoses

The clinical diagnosis of Gitelman syndrome remains largely a diagnosis of exclusion, as many nonhereditary conditions can mimic its presentation. Diuretic abuse mirrors salt wasting and also causes high urinary sodium and chloride losses with a ratio of 1 [6].

Several medications can mimic the biochemical pattern of Gitelman syndrome by disrupting electrolyte transport in the kidneys or gastrointestinal tract. For example, proton pump inhibitors can lead to isolated hypomagnesemia, while aminoglycoside antibiotics such as gentamicin are known to temporarily cause a combination of hypokalemia, hypomagnesemia, and metabolic alkalosis, effects that typically resolve within two to 6 weeks after discontinuing the drug [22–24].

Gastrointestinal losses are another important category to consider. Laxative abuse leads to the depletion of potassium and magnesium through the digestive tract, which results in low urinary sodium and chloride, along with reduced renal fractional excretion of these electrolytes [25]. In contrast, repeated vomiting, such as in bulimia nervosa, can also cause hypokalemia and metabolic alkalosis, but it is often characterized by low urinary chloride levels and a high urinary sodium-to-chloride ratio [26].

Diuretic abuse, though sometimes difficult to detect, can be uncovered by measuring how urinary chloride levels vary during the day. Confirmatory testing with a urinary diuretic screen can provide more definitive evidence [27, 28].

Lastly, primary hyperaldosteronism should be considered, especially when hypokalemia and metabolic alkalosis are accompanied by hypertension and low plasma renin activity. These features help distinguish it from other normotensive conditions with similar electrolyte disturbances [29, 30].

When a genetic cause of tubulopathy is being considered, it is important to remember that Gitelman syndrome is not the only possibility, several other conditions can look very similar. For instance, mutations in the *CLCNKB* gene, which causes Type 3 Bartter syndrome, or in *KCNJ10*, responsible for EAST (or SeSAME) syndrome, can result in nearly identical lab findings. However, EAST syndrome usually comes with clear neurological symptoms such as epilepsy or ataxia, which can help set it apart [31, 32]. *HNF1B* mutations can mimic Gitelman syndrome, but features such as early kidney issues and a family history of diabetes or cysts help differentiate them. Genetic testing is crucial for accurate diagnosis [33, 34].

### 4.5. Value of Genetic Testing

#### 4.5.1. Advantages of Genetic Testing

Diagnosing Gitelman syndrome can be challenging due to overlapping conditions and highly variable symptoms, even among relatives. Some individuals may have significant, long-term issues, while others remain entirely symptom-free [21]. Confirming biallelic *SLC12A3* mutations through genetic testing provides a definitive diagnosis, supports family screening, and helps patients better understand and accept their condition [35].

#### 4.5.2. Challenges of Genetic Testing

A subset of patients exhibiting clinical and biochemical signs consistent with Gitelman syndrome have been found to carry only monoallelic mutation in the *SLC12A3* gene [36]. The prevalence of such heterozygotes is relatively high, about 1% in Caucasians and up to 3% in some Chinese populations. Many patients remain undiagnosed due to limited access to genetic testing. Therefore, rapid and affordable testing for *SLC12A3*, *CLCNKB*, *HNF1B*, and *KCNJ10* is essential. Next-generation sequencing, including whole exome and genome approaches, offers an effective way to establish a genetic diagnosis [21, 34].

A common factor in all the cases in our series is the absence of genetic testing to confirm the diagnosis, which constitutes a major challenge. Although clinical and biochemical signs are suggestive, genetic testing would have confirmed the diagnosis and further personalized the treatment. The absence of this test is attributable to cost and access constraints, a frequently encountered issue in clinical practice [37].

##### 4.5.2.1. Therapeutic Management of Gitelman Syndrome

**Figure 1:**
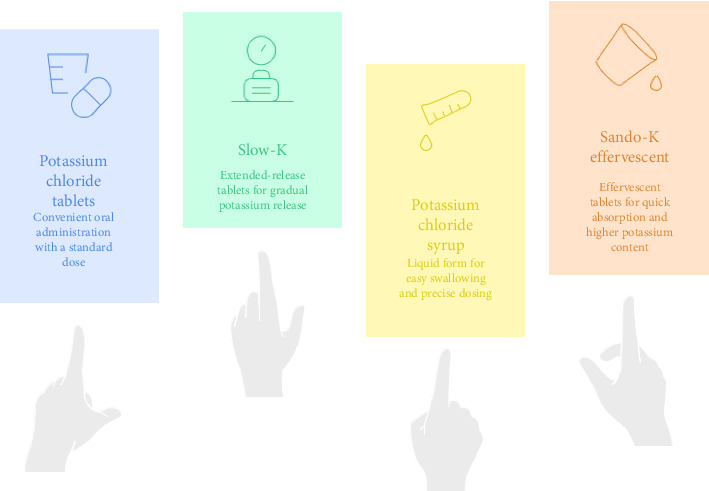
Different potassium treatment preparations [34].

The primary treatment for Gitelman syndrome involves lifelong supplementation with potassium, magnesium, and sodium, with various potassium formulations available depending on patient tolerance and needs (Figure 1) [38]. Alongside dietary advice, long-term intake of potassium and magnesium supplements is recommended (Table 2).

Correcting electrolyte imbalances often remains challenging and may require repeated adjustments in treatment [34].

In the presence of hypomagnesemia, magnesium should be corrected first to enhance potassium repletion. Recommended thresholds are potassium > 3 mmol/L and magnesium > 0.6 mmol/L, but these should be adjusted to the patient's clinical context [39].

Although the minimal serum potassium and magnesium concentrations required to prevent sudden cardiac death remain unknown, maintaining these electrolytes within normal ranges is generally considered safer. Overdosing is rare unless chronic kidney disease is present [26]. Treatment should focus on relieving symptoms rather than strictly correcting electrolyte levels. While magnesium supplementation shows no proven benefit in patients with normal levels, a trial may be considered if symptoms continue [18, 34]. This approach allows for individualized symptom control without unnecessary overtreatment.

Chronic volume depletion is another important concern that must be addressed to improve clinical outcomes. In this context, sodium replacement therapy holds potential therapeutic value. Although no clinical trials have yet confirmed its benefits in Gitelman syndrome, a liberal sodium intake, supplemented with sodium chloride tablets, is often recommended [40]. This strategy aligns with the pathophysiology of renal salt wasting and is supported by frequent reports of salt craving in these patients. Administering sodium chloride tablets may enhance renal sodium load, suppress renin activity, mitigate metabolic alkalosis, and replenish chloride. These effects could reduce potassium requirements and their associated side effects, thereby improving quality of life [6].

##### 4.5.2.2. Challenges of Electrolyte Supplementation

Treatment and supplementation needs for potassium and magnesium in Gitelman syndrome vary widely among patients, requiring individualized dosing that often does not fully normalize lab values. Oral supplements can be difficult to tolerate, especially magnesium, which frequently causes diarrhea at higher doses. In addition, relying on diet for electrolyte intake is often impractical, as many potassium- or sodium-rich foods are also high in sugar and calories [40].

Although intravenous electrolyte therapy can provide temporary symptom relief in Gitelman syndrome, its effects are often short-lived and limited by patient comfort and access to IV treatment [41]. During episodes of vomiting or diarrhea, patients should be informed that their potassium and magnesium needs will increase, and emergency care may be necessary for severe symptoms [42]. A serum potassium drop of 1 mmol/L reflects a body loss of approximately 100–200 mmol, and levels below 2 mmol/L often warrant intravenous replacement [43]. Electrolyte demands also rise significantly during pregnancy, and aggressive oral or intravenous supplementation has been shown to support healthy maternal and fetal outcomes [44].

##### 4.5.2.3. What Oral Electrolyte Preparations Are Best Tolerated?

In the management of Gitelman syndrome, potassium should primarily be replaced using potassium chloride, as chloride is the predominant anion lost in urine. Depending on patient tolerance and preference, potassium chloride can be administered as effervescent tablets, syrups, or slow-release formulations such as “Slow-K,” which are generally better tolerated [45]. An initial dose of at least 40 mmol/day, divided throughout the day, is typically recommended, with further adjustments based on clinical symptoms, laboratory findings, and gastrointestinal side effects [38].

Magnesium supplementation follows a similar individualized approach. Various formulations are available, including magnesium aspartate, citrate, and lactate, which offer better gastrointestinal absorption. Magnesium chloride may also be considered to replenish concurrent chloride losses. Modified-release preparations, such as magnesium lactate, are preferred due to improved tolerability and reduced gastrointestinal side effects [6]. These are often taken with meals to improve absorption and comfort. An initial dose of at least 12 mmol/day is generally advised, with dosage titrated according to patient symptoms and serum levels [46].

Overall, maintaining electrolyte balance with patient-tailored potassium and magnesium replacement regimens is critical for symptom control and preventing complications in Gitelman syndrome.

##### 4.5.2.4. Our Series

Treatment strategies for these patients included potassium and magnesium supplementation, adjusted according to clinical response and side effects.

• Case Study 1: The treatment included effervescent potassium chloride tablets and close monitoring to adjust doses based on symptoms and biochemical results.
• Case Study 2: Initial potassium supplementation was repeatedly increased to address symptoms of paresthesia, with a preference for slow-release preparations.
• Case Study 3: Treatment focused primarily on correcting potassium levels with rigorous serum monitoring and treatment adjustments to avoid complications.

##### 4.5.2.5. Are There Other Options for Treating Gitelman Syndrome?

Nonsteroidal anti-inflammatory drugs such as indomethacin can raise potassium in Gitelman syndrome but are limited by side effects, so they are generally not recommended except in cases with joint pain [6]. Potassium-sparing diuretics such as amiloride and eplerenone are safer alternatives, offering moderate improvement in potassium levels and metabolic alkalosis, though they require monitoring for volume depletion [47].

##### 4.5.2.6. What Monitoring do Gitelman Syndrome Patients Require?

Patients with Gitelman syndrome should have annual or biannual follow-ups at specialized centers, with more frequent blood tests based on clinical stability. Complications such as chronic kidney disease, Type 2 diabetes, hypertension, and proteinuria should be monitored collaboratively by specialists and primary care [48]. Promoting self-management is essential, as patients should be empowered to track their lab results, adjust medications when appropriate, and recognize treatment side effects [49].

## 5. Conclusion

Gitelman syndrome is a rare inherited renal tubulopathy characterized by electrolyte imbalances, including hypokalemia, hypomagnesemia, and hypochloremic metabolic alkalosis and muscle-related symptoms. These anomalies result from mutations in the *SLC12A3* gene, which encodes the thiazide-sensitive NCCT in the DCT of the kidneys.

The clinical manifestations of Gitelman syndrome often include chronic fatigue, muscle weakness, cramps, and in severe cases, periodic paralysis episodes related to profound hypokalemia. Diagnosis relies on identifying these symptoms in conjunction with specific biochemical findings, such as persistent hypokalemia (potassium < 3 mEq/L), hypomagnesemia, and hypochloremic metabolic alkalosis [50]. Low urinary calcium excretion helps distinguish Gitelman syndrome from other similar disorders, such as Bartter syndrome. Genetic tests confirming mutations in the *SLC12A3* gene may be necessary, especially in atypical cases.

The management of Gitelman syndrome focuses on correcting electrolyte imbalances. This includes supplementation with potassium and magnesium, often supplemented with potassium-sparing diuretics such as spironolactone or amiloride to reduce urinary potassium losses. A sodium-rich diet is also recommended to compensate for urinary sodium losses. Early diagnosis and appropriate management are crucial to prevent severe complications, improve patients' quality of life, and reduce repeated hospitalizations due to severe electrolyte imbalances.

In our case series, we studied three patients with Gitelman syndrome. All patients were treated with oral potassium and magnesium supplements and adjusted according to individual needs based on serum electrolyte levels. Regular follow-up and personalized treatments were crucial to stabilize their condition and prevent severe symptoms. It is noteworthy that genetic testing was not performed in these patients, highlighting the challenges in accurately diagnosing Gitelman syndrome in resource-limited settings.

In conclusion, Gitelman syndrome requires a comprehensive diagnostic and therapeutic approach. Effective management of Gitelman syndrome relies on rigorous clinical and biochemical evaluations, as well as therapeutic interventions tailored to the specific needs of the patients. Our clinical cases illustrate the importance of this personalized approach and the challenges encountered in managing this rare pathology.

## Figures and Tables

**Table 1:** Clinical and biochemical characteristics of the three cases.

Case	Clinical presentation	Initial potassium (mmol/L)	Magnesium (mmol/L)	Other biochemical features
1	Vomiting, general health deterioration	1.0 ⟶ 3.9 (after treatment)	0.6 (severe hypomagnesemia)	Fluctuating K+ levels
2	Paralytic hypokalemia, paresthesia	2.1 ⟶ 3.5 (with treatment)	Not specified	Metabolic alkalosis (HCO_3_^−^ = 31.5 mmol/L)
3	Previously diagnosed, symptomatic relapse after K+ reduction	4.2 (postsupplementation)	Not specified	Creatinine: 88 μmol/L; fluctuations in K+

**Table 2:** Different magnesium treatment preparations.

Type	Form	Route of administration	Concentration	Dose	Magnesium treatments
Lactate of magnesium	Tablet	Oral	84 mg (7 mmol) per tablet	1 tablet	Magnesium L-lactate dihydrate
Magnesium oxide	Capsule/tablet	Oral	84.5 mg (7 mmol) per capsule or 240 mg (20 mmol) per tablet	1 capsule/tablet	Magnesium oxide
Magnesium aspartate	Powder sachet	Dissolved in water	243 mg (10 mmol) per sachet	1 sachet	Magnesium aspartate
Magnesium glycerophosphate	Chewable tablet	Oral (chewable)	97 mg (4 mmol)	1 tablet	Magnesium glycerophosphate
Magnesium gluconate	Tablet	Oral	27 mg (2 mmol)	1 tablet	Magnesium gluconate

## Data Availability

The data supporting the findings of this study are available from the corresponding author upon reasonable request. Due to privacy concerns and the small number of cases, individual patient data cannot be made publicly available.
